# Pathogen-Derived Nucleases: An Effective Weapon for Escaping Extracellular Traps

**DOI:** 10.3389/fimmu.2022.899890

**Published:** 2022-07-05

**Authors:** Chengshui Liao, Fuchao Mao, Man Qian, Xiaoli Wang

**Affiliations:** ^1^College of Animal Science and Technology/Luoyang Key Laboratory of Live Carrier Biomaterial and Animal Disease Prevention and Control, Henan University of Science and Technology, Luoyang, China; ^2^Animal Diseases and Public Health Engineering Research Center of Henan Province, Luoyang Vocational and Technical College, Luoyang, China; ^3^School of Basic Medical Sciences, Henan University of Science and Technology, Luoyang, China

**Keywords:** immune evasion, innate immune cells, extracellular traps, pathogens, nucleases

## Abstract

Since the 2004 publication of the first study describing extracellular traps (ETs) from human neutrophils, several reports have shown the presence of ETs in a variety of different animals and plants. ETs perform two important functions of immobilizing and killing invading microbes and are considered a novel part of the phagocytosis-independent, innate immune extracellular defense system. However, several pathogens can release nucleases that degrade the DNA backbone of ETs, reducing their effectiveness and resulting in increased pathogenicity. In this review, we examined the relevant literature and summarized the results on bacterial and fungal pathogens and parasites that produce nucleases to evade the ET-mediated host antimicrobial mechanism.

## Introduction

Pathogens have evolved various strategies to overcome the killing mechanisms of the host immune system and establish a productive infection. The secreted proteins of pathogens, which include proteases, lipases, collagenases, hyaluronidases, chitinases, and nucleases, have been found to be essential for evading the host defense system (1). Nucleases are a diverse group of enzymes that degrade both DNA and RNA, and there are several ways to classify them (2). According to Linn formulated the criteria, the most acceptable system for classification of nucleases is to divided them into sugar specific and sugar non-specific nucleases (3). Based on their activity on ester bonds, nucleases are considered a subgroup of hydrolases and can be subdivided into exonucleases (EC 3.1.11.X to EC 3.1.16.X) and endonucleases (EC 3.1.21.X to EC 3.1.31.X). For the ability to hydrolyze DNA, sugar specific nucleases are DNases including exodeoxyribonucleases and endodeoxyribonucleases, while sugar non-specific nucleases comprise endonucleases and exonucleases (2). According to their biochemical and biological properties as well as tissue-specific production, DNases can be further divided into the DNase I and DNase II families (4). Pathogen nucleases are mainly involved in physiological processes such as genetic transformation and nutrient scavenging, DNA replication, recombination and DNA repair mechanisms (5); but, they can also act as virulence factors (5), regulate of biofilm formation, enhance fitness and bacterial shedding (6), and allow microbes to enter non-phagocytic cells or survive and replicate in phagocytic cells.

**Figure 1:**
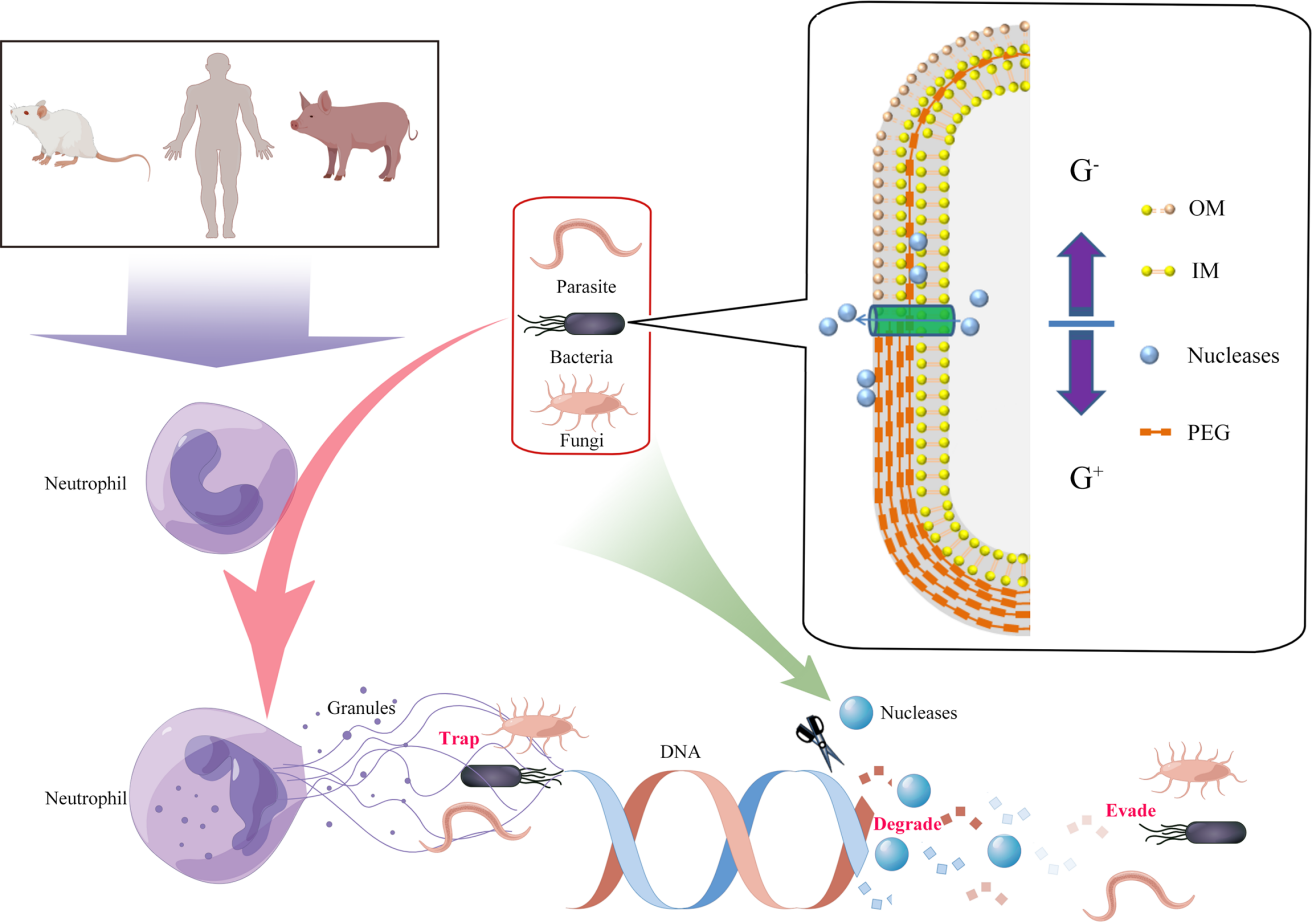
Degradation of extracellular traps (ETs) by the production of pathogen-derived nucleases. Innate immune cells were activated by pathogens, and DNA from nuclei or mitochondria mixed with histones, granular, and cytoplasmic proteins was released to the cellular environment (termed ETs). ETs could immobilize and/or kill the pathogens. However, secreted or cell wall­anchored extracellular nucleases of pathogens were capable of destroying DNA backbones of ETs and contributing to evasion of the immune response and development of persistent infections. G^+^, Gram-positive bacteria; G^-^, Gram-negative bacteria; OM, outer membrane; IM, inner membrane; PEG, peptidoglycan.

Neutrophils are the most abundant type of white blood cell, and they play an important role in inflammation and host defense through the respiratory burst, degranulation, and phagocytosis (7). In 2014, Brinkmann et al. reported that when attacked by pathogens, human neutrophils released DNA-based network structures called neutrophil extracellular traps (NETs), which immobilized and/or killed pathogens (8). This phenomenon has also been observed in different mammalian species, birds, fish, invertebrates, and plants (9). However, it is also known that certain pathogens have developed strategies to avoid the killing effects of extracellular traps (ETs) by secreting a polysaccharide capsule, forming biofilms, undergoing cell surface modifications, and inhibiting ET formation (10). The main components of ETs are granule proteins and DNA, therefore it was predicted that extracellular nucleases could directly degrade the ETs’ scaffold DNA, providing an effective way for pathogens to escape the ET defensive network structures (**Figure 1**). In this article, we review the latest research on the role of extracellular nucleases of bacteria, fungi, parasites, and mycoplasmas in degrading the backbone DNA of ETs, especially NETs (**Table 1**).

**Table 1:** Pathogen-derived nucleases that degrade ETs.

Pathogene	Nuclease	Nuclease activity	Cellular localization	ET degradation*	Conserved motifs	Reference
Vibrio cholerae	Xds	Exonuclease	Membrane anchored	Deletion mutant increased PMA-inducted NETs in human neutrophils.	LTD, OBD, EEPD	(11–13)
	Dns	Endonuclease	Extracellular secreted	Deletion mutant increased PMA-inducted NETs in human neutrophils.	ND	(11, 12)
Streptococcus pneumoniae	EndA	Endonuclease	Membrane anchored	Deletion mutant failed to degrade NETs induced by PMA or H_2_O_2_ in human neutrophils *In vivo*: NETs localized to alveoli in deletion mutant-infected lungs of C57BL/6 mice.	DRGH, HNH(N)	(14–20)
	TatD	Exo/endonuclease	Cell-wall surface anchored	rTatD expressed in E. coli degraded PMA-inducted NETs in human neutrophils, but little degrading activity in deletion mutant.	ND	(21)
Group A Streptococcus	Sda1	DNase	Cell-wall anchored	Significant quantities of NETs persisted in human neutrophils infected with deletion mutant; Sda1-expressing L. lactis eliminated NETs in human neutrophils. *In vivo*: NETs were clearly visualized in murine skin abscess model injected with deletion mutant.	LPXTG, DRSH	(22)
Streptococcus pyogenes	SpnA	Exo/endonuclease	Cell-wall anchored	Deletion mutant increased PMA-inducted NETs in human neutrophils; rSpnA expressed in E. coli and SpnA-expressing L. lactis cleaved PMA-inducted NETs in human neutrophils.	LPXTG, EEPD, OBD	(23, 24)
Streptococcus suis	SsnA	DNase	Cell-wall anchored	Deletion mutant attenuated NET degradation in PMA-stimulated human neutrophils, but not porcine.	LPXTG	(25)
	EndAsuis	Endonuclease	Membrane anchored	Deletion mutant attenuated NET degradation in PMA-stimulated human neutrophils during exponential growth, but not in stationary phase.	DRGH	(26)
Streptococcus equi subsp. zooepidemicus	ENuc	Sugar non-specific nuclease	Cell-wall anchored	rENuc expressed in E. coli degraded PMA-inducted NETs in neutrophils of ICR mice.	LPXTG, sugar-nonspecific nuclease domain, EEPD	(27)
	5Nuc	5’-nucleotidase	Cell-wall anchored	r5Nuc expressed in E. coli degraded PMA-inducted NETs in neutrophils of ICR mice.	LPXTG, 5’-nucleotidase domain	
Streptococcus agalactiae	NucA	Exo/endonuclease	Extracellular secreted	The nucA H148A mutant had no effect on NET response in PMA-activated mice neutrophils.	DKGH, HNN	(28, 29)
Streptococcus mutans	DeoC	Nuclease	ND	Deletion mutant stimulated NET production in human neutrophils.	ND	(30)
Streptococcus sanguis	SWAN	Sugar non-specific nuclease	Cell-wall anchored	rSWAN expressed in E. coli digested PMA-inducted NETs in human neutrophils	LPKTG, EEPD, OBD	(31)
Streptococcus iniae	SpnAi	ND	ND	rSpnAi expressed in E. coli degraded PMA-inducted NETs in neutrophils from adult zebrafish kidney.	ND	(32)
Staphylococcus aureus	Nuc1/SNase	Sugar non-specific nuclease	Extracellular secreted	Deletion mutant showed impaired degradation of PMA-inducted NETs in human neutrophils; Snase-expressing L. lactis cleaved PMA-induced NETs in neutrophils of NOD mice.	ND	(33–39)
Mycobacterium tuberculosis	Rv0888	Exo/endonuclease, Phosphatase, Smase	Extracellular secreted/ Membrane anchored	The Smase activity of Rv0888 efficiently induced NET formation in human neutrophils, but not nuclease activity.	ND	(40–42)
Pseudomonas aeruginosa	EddB	DNase, PDE	Extracellular secreted	The EddAB double mutant with plasmid-encoded pEddB restored the NET degradation capacity in human neutrophils.	ND	(43)
Neisseria gonorrhoeae	Nuc	Sugar non-specific nuclease	Extracellular secreted	rNuc expressed in E. coli decreased the integrity of PMA-induced NETs in human neutrophils, as well as NETs elicited by the deletion mutant.	ND	(44)
Prevotella intermedius	nucA	Sugar non-specific nuclease	Extracellular secreted	rNucA expressed in E. coli was capable of cleaving PMA-induced NETs in human neutrophils.	EEPD	(45)
	nucD	Sugar non-specific nuclease	Extracellular secreted	rNucD expressed in E. coli was capable of cleaving PMA-induced NETs in human neutrophils.	Endonuclease_NS domain	
Ralstonia solanacearum	NucA/NucB	Endonuclease	Membrane anchored	The nucA/B deletion mutant was immobilized by the DNA of plant border cell traps, and reversed by rNucA and rNucB expressed in E. coli.	ND	(46)
Leishmania spp.	3’NT/NU	Sugar non-specific nuclease	Cell surface membrane-anchored	LP parasites with higher 3’NT/NU activity cleaved more NETs in buffy coat neutrophils.	Class I nucleases domain	(47–49)
Nippostrongylus brasiliensis	Nb-DNase	DNase II	ND	The extracellular DNA fibers formed in human neutrophils induced by PMA were hydrolyzed by rNb-DNase II expressed in E. coli.	DNase II	(50)
Plasmodium spp.	PbTatD	DNase	Parasitophorous vacuole membrane	The deletion mutant induced METs and NETs in J774A.1 macrophages and mouse neutrophils, but fewer in WT parasites; rPbTatD expressed in E. coli hydrolyzed METs.	DNase	(51, 52)
Trypanosoma spp.	TryTatD05/TryTatD15	Endonuclease	Cytoplasm, flagella	rTryTatD05 and rTryTatD15 expressed in E. coli degraded NETs in murine neutrophils.	HPEDHRHFGEDP	(53)
Mycoplasma hominis	MHO_0730	Sugar non-specific nuclease	Membrane anchored	Viable Mycoplasma hominis degraded PMA-induced NETs in human neutrophils.	TNASE_3 domain	(54, 55)
Mycoplasma hyopneumoniae	Mhp597	Sugar non-specific nuclease	Extracellular secreted	PMA-induced NETs in porcine were completely destroyed by rMhp597 expressed in E. coli.	ND	(56)
Mycoplasma bovis	MBOV_RS02825	Sugar non-specific nuclease	Membrane anchored	rMBOV_RS02825 expressed in E. coli reduced PMA-induced NETs in bovine neutrophils.	TNASE_3 domain	(57)
	MnuA	ND	Membrane anchored	NETs were evident in cow neutrophils stimulated with the deletion mutant.	ND	(58)
Mycoplasma pneumoniae	Mpn491	Nuclease	Extracellular secreted	The ability of the deletion mutant to degrade PMA-induced NETs in human PMNs was markedly impaired. *In vivo*: LPS-induced NETs in mice lungs were apparently released by the infection with the deletion mutant.	EEPD	(59)
Leptospira spp.	LigA	Exo/endonuclease	Surface Protein	rLigA expressed in E. coli degraded the PMA-induced NETs in neutrophils of C57BL/6J mice.	ND	(60)

*Conditions under which pathogen-derived nucleases degrade ETs in vitro and in vivo.

ND, not determined; ETs, extracellular traps; NETs, neutrophil extracellular traps; PMA, phorbol myristate acetate; Smase, sphingomyelinase; PDE, phosphodiesterase; LTD, lamin-tail domain; OBD, oligonucleotide/oligosaccharide-binding fold domain; EEPD, endonuclease/exonuclease/phosphatase domain.

## Bacteria

### Vibrio cholerae

*Vibrio cholerae* is a Gram-negative comma-shaped bacterium that is the causative agent of the secretory diarrheal disease cholera. Extracellular DNA is a major component of the *Vibrio* biofilm matrix. The two nucleases secreted by *V. cholerae*, Xds and Dns, are able to degrade extracellular DNA. Dns exhibits endonuclease activity, while Xds has been characterized as an exonuclease (11). Extracellular DNA degradation by the two nucleases results in accumulation of high levels of nucleotides outside the cell. OmpK was used to transport nucleotides to the outer membrane, where they were dephosphorylated by periplasmic phosphatases. Free phosphate was absorbed by the Pst/PhoU system and nucleosides were transported into cells *via* NupC (61). *V. cholerae* exposure caused human neutrophils to form NETs, while secreted Dns and Xds rapidly degraded the NETs’ DNA and released the bacteria from the network structures, thus promoting cell colonization by *V. cholerae* (12). Infection with a *Dns/Xds* deletion mutant of *V. cholerae* resulted in prolonged stability of the NET. The C-terminus of Xds has an exonuclease domain, and residues D787 and H837 within the exonuclease domain are the key sites of the catalytic center (13).

### *Streptococcus* spp.

#### Streptococcus pneumoniae

The endonuclease, EndA, of *S. pneumoniae* is localized on the membrane surface and is a key factor in DNA replication and bacterial virulence. EndA is required for the genetic transformation of *S. pneumoniae* and contributes to gene transfer and genetic diversification (14–16). EndA cannot bind to external DNA or degrade it when *S. pneumoniae* does not undergo gene conversion (17). However, in another study, it was reported that degradation of extracellular DNA by EndA did not require components of the competence regulon (18). EndA was secreted extracellularly during *S. pneumoniae* growth, which helped the pathogen establish an infection in the lung by degrading the DNA backbone of NETs and overcoming the host immune system (18). EndA possesses a DRGH (Asp-Arg-Gly-His) motif containing a ββα metal-finger catalytic core, and the amino acids, His160, Asn191, and Asn182 that are essential for catalytic activity. Expression of the wild-type *EndA* gene in *E. coli* was difficult due to its extreme cytotoxicity, but an *EndA* gene with the H160A mutation was successfully expressed. Asn182 was required for the stability of EndA (19), while Arg127/Lys128 and Arg209/Lys210 helped the enzyme bind to the substrate (20). In addition, the three mutant strains, H154A, Q186A, and Q192A, exhibited significantly decreased nuclease activities.

The twin-arginine translocase D (TatD) is a soluble cytoplasmic protein that was obtained from the extracellular secreted proteins of *S. pneumoniae*, but has been described in most prokaryotic and eukaryotic species (21). Although TatD functions primarily in the operation of the Tat transport system, it also exhibits endonuclease and exonuclease activity. TatD has been associated with the formation of extracellular vesicles of *S. pneumoniae*, and recombinant TatD (rTatD) had DNase activity. A *TatD* deletion mutant showed little NET degradation activity, while the addition of rTatD reduced the formation of NETs. The deletion mutant greatly reduced the bacterial load in the lung, blood, and spleen in a murine sepsis model (21). In our study, the recombinant TatD-like DNase from *Listeria monocytogenes* 10403s was associated with the degradation of macrophage ETs and its Mg^2+^-dependent nuclease activity had an optimum reaction temperature of 37°C and pH of 6.0 (62).

#### *Streptococcus pyogenes* (GAS, group A streptococci)

Group A streptococci (GAS) produce 26-30 extracellular nucleases, including Sda1, SdaD2, Spd, and Spd3. Sda1 exhibits cell wall-anchored DNase activity and highly expressed by the serotype M1 GAS strain. Sda1 contributed to enhancing the tolerance and virulence of GAS toward neutrophils in a murine model of necrotizing fasciitis. Inhibition of GAS nuclease activity with G-actin enhanced the clearance of pathogens by neutrophils *in vitro* and reduced virulence *in vivo* (63). Sda1 from invasive M1T1 GAS degraded the DNA backbone of NETs in human neutrophils. DNA degradation by Sda1 prevented GAS from inducing murine macrophages to secrete IFN-α and TNF-α, while the levels of IFN-α and TNF-α in mice were significantly decreased by *Sda1*-expressing GAS (64). Degradation of PMA-induced NETs by Sda1 and other phage-encoded DNases in mouse neutrophils was neutralized by antibodies from mice immunized with Sda1 (22). The prophage-encoded SdaD2 enzyme was the major DNase that contributes to GAS virulence (65). Extracellular killing of the *spd3*/*sdaD2*/*spd* deletion mutant of M1 GAS strain MGAS5005 in human PMNs was significantly enhanced. In addition, the virulence of the triple-mutant strain in mice was significantly reduced and bacteria were easily removed from the skin injection site (65).

The *Streptococcus pyogenes* nuclease A (SpnA) is an exo/endonuclease with an LPXTG motif located on the cell surface in *S. pyogenes* and also GAS. Two catalytic domain structures were predicted for the mature SpnA. Multiple oligonucleotide and oligosaccharide binding-fold motifs were found in the N-terminal domain at position 99-395. The C-terminal domain contained a putative endo/exonuclease domain at position 549-851. Glu592, Arg696, His716, Asp767, Asn769, Asp810, and Asp842 were necessary for SpnA activity and contributed to binding of the substrate (23). Both the full-length (99-877) and the truncated (217-877) versions of the recombinant SpnA expressed in *E. coli* exhibited nuclease activity. The rSpnA was capable of dismantling the NET-like structures produced by human neutrophils after stimulation with phorbol myristate acetate (PMA) (24). The nuclease activity of the *spnA* deletion mutant was decreased by about 70%, and antibodies against SpnA in human serum significantly inhibited nuclease activity. Although SpnA promoted bacterial survival and neutrophil killing in whole human blood, the *spnA* deletion mutant showed only a partial reduction in virulence in a *Galleria mellonella* infection model (23).

5′-nucleotidases catalyze the hydrolysis of phosphate esterified at carbon 5′ of ribonucleotides, deoxyribonucleotides, and complexnucleotides (66). 5′-nucleotidases are allocated to the enzyme commission numbers EC 3.1.3.5, and are found in all kingdoms, including plants, animals, bacteria, fungi, and parasites. The biological function of microbial 5′-nucleotidases is related to the location of the enzyme in the cell (66). Membrane-bound or cell wall-anchored 5′-nucleotidases has the ability to convert AMP into the immunomodulator adenosine, thus contributing to the evasion of the bacteria from the host immune response during infection (67). *Streptococcal* 5’-nucleotidase A (S5nA) is a cell wall-anchored 5′-nucleotidases and also a virulence factor of *S. pyogenes* N99A. Recombinant S5nA (rS5nA) produced in *E. coli* showed 5′-nucleotidase activity that hydrolyzed AMP, ADP, and dAMP, but not ATP, to generate adenosine and deoxyadenosine. Expression of rS5nA increased the survival of non-pathogenic *Lactococcus lactis* in human whole blood (68). However, the current data are still not sufficient to prove the role of S5nA in immune evasion by degrading the DNA backbone of ETs.

Streptococcal phage-encoded DNase (Spd1) is a type I extracellular DNase with 28 kDa encoded by a prophage (SF370.1) in *S. pyogenes* strain SF370. Spd1 existed as a monomer in solution and His121, Asn145, and Glu164 were the important conserved residues for nuclease activity (69). Although phage-encoded DNases are capable of promoting bacterial and phage particle dissemination by liquefying pus and cellular material and enhanced the survival of the phage, the crucial role of Spd1 in *S. pyogenes* in evading the host immune response is not fully understood.

#### Streptococcus suis

SsnA is a secreted DNase that is anchored in the cell wall of *S. suis* and released into the culture medium. SsnA had strong DNase activity during exponential growth. The *ssnA* deletion mutant significantly reduced bacterial adherence and invasion of human laryngeal epithelial Hep-2 cells, and the virulence of the *S. suis* deletion mutant in CD1 mice was significantly decreased (70). SsnA was not necessary for *S. suis* type 2 growth and survival in human and porcine blood *in vitro*, but it did contribute to degradation of NETs stimulated by PMA in human and porcine neutrophils and weakened the antibacterial activity of human NETs (25).

EndAsuis is an endonuclease of *S. suis* with high homology and structural similarity to the membrane anchor of EndA in *S. pneumoniae*, but EndAsuis could not be successfully expressed in *E. coli* (26). The nuclease activity of a recombinant EndAsuis with a point-mutation at H165 in the DRGH motif was confirmed in the presence of Mg^2+^, but not Ca^2+^. EndAsuis was not released from *S. suis* into the culture medium; however, the *EndAsuis* deletion mutant significantly attenuated the degradation of human NETs, but had no significant effect on the NET-mediated bactericidal activity (26). High DNase activity of SsnA was found in the cerebrospinal fluid (CSF) of *S. suis*-infected piglets with severe suppurative meningitis. However, neither SsnA nor EndAsuis efficiently degraded NETs, and NET fibers with entrapped streptococci were observed in neutrophils from CSF (71). The up-regulated cathelicidin PR-39 in choroid plexus epithelial cells inhibited the degradation of NETs. In contrast to SsnA, host DNase 1 contributed to the enhancement of neutrophil antimicrobial activity in the CSF of *S. suis*-infected piglets (72).

#### *Streptococcus equi* subsp. *zooepidemicus*

ENuc and 5Nuc, encoded by the *SESEC_RS04165* and *SESEC_RS05720* genes, respectively, are two extracellular nucleases from *S. equi* subsp. *zooepidemicus*. ENuc and 5Nuc were a cell-wall anchored nucleotidase with LPXTG motif. The degradation of NETs by these nucleases reduced phagocytosis and the bactericidal capacity of macrophages, but not the intracellular killing process. An *Enuc*/*5Nuc* deletion mutant lost the ability to degrade NETs into deoxyadenosine, but it induced fewer NETs and showed greater survival in the NETs that were produced (27).

#### Streptococcus agalactiae

Seven genes encoding secreted nucleases from *S. agalactiae* have been found. NucA encoded by the *gbs0661* gene displays a high degree of sequence identity with *S. pneumoniae* EndA and *S. pyogenes* Sda1. NucA contains a putative N-terminal transmembrane domain and is a major extra-cytoplasmic nuclease that required divalent cations, a stable pH, and heat stability. The nuclease activity of the H148A, R111A/H148G, K127A/H148G, K146A/H148G, and Q180A/H148G point-mutated strain was significantly decreased, while the K146R and Q183A mutant strain exhibited significantly increased activity (28). NucA was required to avoid *S. agalactiae* clearance from lung tissue at an early step of infection by degrading the DNA skeleton of NETs induced by PMA in mice, and contributed to dissemination in the bloodstream and persistent infection (29).

#### Streptococcus mutans

The *S. mutans* culture medium at 48-60 h exhibited a high level of DNase activity, which changed with the same tendency as the biofilm-released bacterial cells. The *deoxyribose aldolase* gene (*deoC*)-encoded product catalyzed the reversible aldol reaction of acetaldehyde and glyceraldehyde 3-P from the sugar phosphate, deoxyribose 5-phosphate (73) and also catabolized extracellular DNA. The nuclease DeoC was a regulator of the biofilm dispersal of *S. mutans*. *S. mutans* induced the formation of NETs in human neutrophils, and the NETs in turn enhanced the expression of the *deoC* gene of *S. mutans*. The DeoC activity of *S. mutans* was critical for evading killing by neutrophils through degrading NETs (30).

#### Streptococcus sanguis

Morita et al. reported that oral streptococci showed extracellular DNase activity. Thirty-three cell wall-anchored proteins containing the LPXTG motif were predicted in *S. sanguis*. SWAN is a cell surface protein with a cell wall-sorting signal and a putative nuclease domain. The nuclease activity of a *Swan* deletion mutant was significantly reduced, while a strain over-expressing recombinant SWAN degraded a variety of DNAs including the DNA backbone in NETs, allowing *S. sanguis* to survive NETs from human neutrophils stimulated with PMA. In addition, *L. lactis* heterologously expressing *Swan* showed enhanced resistance to NET-mediated bactericidal activity (31).

#### Streptococcus iniae

The genes encoding SpnAi and S5nAi in *S. iniae* were similar to those for SpnA and S5nA of GAS, respectively, in terms of amino acid sequence, protein length, domain structures, and biochemical properties, as well as similar virulence mechanisms (74). The *spnAi* and *s5nAi* deletion mutants showed significant loss of DNase and nucleotidase activity, respectively. SpnAi and S5nAi supported the growth, proliferation, and dissemination of *S. iniae* and contributed to *S. iniae* virulence in zebrafish larvae. The *SpnAi* and *S5nAi* deletion mutants were still able to recruit neutrophils and macrophages to infected sites (32). NETs induced by PMA were degraded in the presence of *S. iniae* and also the recombinant SpnAi in neutrophils from the kidneys of adult zebrafish, but not in the presence of the s*pnAi* deletion mutant (32).

### Staphylococcus aureus

Staphylococcal nuclease (SNase) is a nonspecific phosphodiesterase that has a strong ability to degrade a DNA scaffold to ssDNA or dsDNA, and we found that the secreted proteins of *S. aureus* possess DNA degradation activity (75). Similarly, Herzog et al. reported significant nuclease activity of clinical *S. aureus* isolates from cystic fibrosis (CF) patients (33). The nuclease activity of sequential isogenic isolates was increased in a time- and phenotype-dependent manner. Strong DNA degradation capability was observed in sputum samples from one CF patient during a long-term chronic *S. aureus* infection. The isolates with high nuclease activity facilitated *S. aureus* survival from NET-mediated killing by human neutrophils (33).

Nuc1, a thermostable nuclease, catalyzes the hydrolysis of both DNA and RNA. *S. aureus* eDNA maintains the structural stability of the biofilm during bacterial colonization. During the early stage of *S. aureus* biofilm formation, Nuc1 is expressed, which was responsible for infection persistence in a mouse subcutaneous implant model (76). Nuc1 can degrade the DNA scaffold in neutrophil NETs, thereby evading the host immune response (34). The breakdown of NET DNA by *S. aureus* nuclease Nuc1 contributed to the survival and dissemination of biofilm bacteria trapped in NETs and caused persistent infections (35). The ability of the *nuc1* deletion mutant to degrade NETs was significantly impaired, and it was more sensitive to extracellular killing by activated neutrophils (36). Nuc2, another thermostable nuclease of *S. aureus*, was considered to be a cell surface-binding protein with nuclease activity. However, Nuc2 had no significant impact on NETs and was not involved in virulence and immune evasion (37).

*S. aureus* degrades NET DNA into deoxyadenosine to avoid its killing effect. The process triggered the caspase 3-mediated death of immune cells (38). Excessive formation of NETs contributed to the early pathological injury to the pancreas, resulting in the onset of diabetes. Lang et al. showed that a *L. lactis* strain expressing recombinant *SNase* effectively degraded PMA-induced NETs *in vitro* and significantly decreased circulating free DNA in the serum in non-obese diabetic (NOD) mice (39). Moreover, early treatment with SNase ameliorated the gut immune microenvironment of NOD mice by regulating the level of NETs in the intestinal mucosa (77).

### Other Bacterial Species

Three different *Yersinia enterocolitica* serotypes (O:8, O:9, and O:3) were able to induce NET formation. The culture supernatants of the three serotypes also showed the capacity to degrade plasmid DNA in the presence of Ca^2+^/Mg^2+^, and NETs induced by PMA treatment of human neutrophils were degraded by the bacterial supernatants (78). Although the endonuclease-1 proteins, EndA and NucM, of *Y. enterocolitica* are similar to the NET-degrading *V. cholerae* extracellular deoxyribonuclease, their role in degrading the DNA of NETs to evade the host immune response remains unclear.

Three extracellular nucleases, EndA, ExeS, and ExeM were found in *Shewanella oneidensis* MR-1. ExeM was localized to the cytoplasmic membrane fraction. The activity of ExeM expressed in *E. coli* revealed that it was a nonspecific endonuclease requiring Ca^2+^ and Mg^2+^ or Ca^2+^ and Mn^2+^ as cofactors. ExeM was beneficial for biofilm formation under static conditions, however, externally added recombinant ExeM inhibited biofilm formation but not biofilm removal (79). Although the author hypothesized that ExeM may be related to NETs degradation, not detected in culture supernatants of *S. oneidensis*. Thus, the role of ExeM in NET-mediated extracellular killing is still unclear.

Sphingomyelinase catalyzes the hydrolysis of sphingomyelin into ceramide and phosphorylcholine, and is allocated to the enzyme commission numbers, EC 3.1.4.12 and EC 3.1.4.41 (80). More than six categories of sphingomyelinase have been found in a wide variety of bacteria (80, 81). The important physiological functions of sphingomyelinase are related to membrane permeability, membrane aggregation, and fusion (80). Sphingomyelinase as a virulence factor contributes to bacterial colonization, dissemination, and evasion of immune response for the establishment of persistent infection (81). Rv0888 obtained from *Mycobacterium tuberculosis* culture filtrate is an outer membrane protein with sphingomyelinase activity. Rv0888 increased intracellular survival, and replication of *M. tuberculosis* in THP-1 macrophages, but was not required for virulence of *M. tuberculosis* in mice (40). The C-terminal sequence of Rv0888 was highly conserved with an endonuclease-exonuclease-phosphatase domain (40). Dang et al. demonstrated that the recombinant Rv0888 expressed in *E. coli* required divalent metal cations (Ca^2+^ and Mn^2+^) to degrade different types of nucleic acids (41). The optimum temperature and pH for the nuclease activity were 41°C and 6.5, respectively. The activity of Rv0888 was inhibited by four traditional Chinese medicinal compounds, oleuropein, 6-gingerol, corylifolinin, and acteoside. The residues, H353, D387, D438, and H481, were essential for catalysis of Rv0888 (41). Although *M. tuberculosis* induced the formation of NETs and could be captured by them, the bacteria were not killed by NETs *in vitro*. Interestingly, the sphingomyelinase activity of recombinant Rv0888NS/MS stimulated the release of NETs from human neutrophils *in vitro* and in the lung tissues of mice (42).

Phosphodiesterases (EC 3.1.4.1) are a superfamily of enzymes that cleave the cyclic phosphodiester bond of cyclic adenosine and cyclic guanosine monophosphate (82). There are a total of 11 different phosphodiesterase family members (phosphodiesterase 1-11) with 21 isoforms. Phosphodiesterases are involved in diverse physiological functions and play a pivotal role in mediating the cyclic nucleotide signaling (82). In pathogens, phosphodiesterases are essential for controlling the kinetics of biofilm formation, swarming motility, survival, and dissemination (83). *Pseudomonas aeruginosa* has two secreted nucleases, EddA and EddB. EddA has alkaline phosphatase and phosphodiesterase activity, which can restrict the antibacterial activity of NETs (43). EddB can degrade eDNA, and the products can be taken up and used as source of nutrition by cells. The eDNA and NETs were effective inducers of the nuclease-phosphatase operon. The nucleases helped to degrade the DNA backbone of NETs and protected *P. aeruginosa* from NET-mediated killing. Although phosphatase did not the ability of the DNases to degrade DNA, it prevented cations from chelating phosphate from the eDNA phosphodiester backbone. Therefore, both DNase and phosphodiesterase contributed to the resistance to killing of *P. aeruginosa* by NETs (43).

*Neisseria gonorrhoeae* stimulated human neutrophils to release NETs, and the NETs showed antibacterial activity against *N. gonorrhoeae*. A thermostable nuclease homologue, Nuc, from *N. gonorrhoeae* influenced biofilm formation. Recombinant Nuc expressed in *E. coli* degraded DNA in PMA-induced NETs, and enhanced the survival of extracellular bacteria in human neutrophils (44).

Several studies have demonstrated that the NET-like defense structure as a mechanism is analogous to the situation in periodontitis, where bacteria colonize the periodontal region by avoiding the immune response of the host. Doke *et al.* reported that the periodontal pathogenic bacteria, *Porphyromonas gingivalis*, *Prevotella intermedia*, and *Fusobacterium nucleatum* exhibited extracellular DNA degradation activity (84). The genes, *nucA* and *nucD*, and the encoded secreted nucleases were characterized in *P. intermedia* (ATCC 25611). Recombinant NucA and NucD required Mg^2+^ and Ca^2+^ for optimal digestion of DNA and RNA, and their ability to degrade the DNA framework of NETs was confirmed (45). In our present study, the extracellular secreted proteins from *Salmonella typhimurium*, *S. choleraesuis*, *Klebsiella pneumoniae*, and *Pasteurella multocida* showed nuclease activity in degradation of phage λ DNA. Four proteins with nuclease activity were identified as extracellular proteins secreted from *S. Typhimurium* using the SDS-PAGE nuclease zymography technique combined with LC-ESI/MS/MS (85). METs -induced by *Candida albicans* were degraded by secreted proteins from *S. choleraesuis* (86). However, detailed studies are needed to assess the roles of the extracellular secreted proteins in allowing the bacteria to escape from the ETs.

In peas, corn, and cotton, extracellular DNA with histone is a component of the extracellular matrix secreted from the root cap. This structure was found to function like animal NETs to allow root border cells to capture pathogenic bacteria and fungi in the soil and immobilize them (46). Plant root border cells release DNA-containing ETs in response to the high-impact pathogenic plant bacterium, *Ralstonia solanacearum*. Two functional extracellular DNases, NucA and NucB, were identified in *R. solanacearum* strain GMI1000. The *nucA/B* deletion mutant was immobilized by the DNA of plant border cell traps, while the bacterial trapping was reversed by treatment with recombinant NucA and NucB (46).

## Fungi

### *Candida* spp.

3’-nucleotidase/nuclease (3′-NT/NU, EC 3.1.3.6) is a bifunctional enzyme capable of hydrolyzing 3′-monophosphorylated nucleotides and nucleic acids to generate nucleosides and inorganic phosphate. 3′-NT/NU is about 40 kDa and has five highly conserved regions. The activity of 3′-NT/NU was found in a wide variety of species, including fungi, protozoan, plants, and bacteria (87). The nuclease exhibits diverse functions in different species; however, the process of sulfate assimilation is a remarkably conserved property of the core machinery (88). The yeast nucleotide phosphatase, MET22, is closely associated with the incorporation of inorganic sulfates into amino acids, and also plays an important role in cell growth under high concentrations of Na^+^ and Li^+^. Numerous studies have reported that ETs were induced by yeast and hyphal forms of *Candida* spp (89). We have previously shown that METs induced by *C. albicans* showed the ability to trap the microbes, but with limited microbicidal activity (90). It was reported that 20% of *Candida* spp. showed *in vitro* DNase activity (91). *C. albicans* escape NETs through extracellular secretion of DNases that degrade the DNA backbone (92). Over recent years, several extracellular nucleotidases from fungi have been identified (93). 3′NT and 5′NT activities were determined in *C. albicans* and *C. glabrata*, and there was higher 3′NT activity at pH 4 (94). The activity was inhibited by ammonium tetrathiomolybdate (TTM), a 3′NT/NU inhibitor. NET formation and release were prevented through the 3′NT/NU activity of *C. albicans* and *C. glabrata*, and a more pronounced phagocytosis by neutrophils was observed. This feature was restored in the presence of TTM and resulted in better control and elimination of *C. albicans* (94).

### *Paracoccidioides* spp.

Paracoccidioidomycosis is a fungal disease with systemic, progressive, chronic, and endemic infection. The members of *Paracoccidioides* spp. are the etiological agents of mycosis. *P. brasiliensis* induced NET formation by human PMNs *in vitro*, which was involved in extracellular fungicidal action (95). Recently, it was demonstrated that *P. brasiliensis* Pb265 and Pb18 were able to induce different patterns of NET formation (96). Pb265 is a harmless strain while Pb18 is virulent. The Pb18 strain had the ability to produce and release extracellular DNase, which degraded the DNA of NETs induced by the Pb18 strain causing them to be looser and more dispersed. Interestingly, the Pb265 strain did not consistently show DNase activity compared to the virulent strain, and its ability to induce neutrophils to form NETs was greater than that of the Pb18 strain (97).

### Cochliobolus heterostrophus

The ET mechanism against fungal infections is also present in plants. This defensive function was abolished by the addition of DNase I, resulting in 100% incidence of root rot. The maize pathogen, *C. heterostrophus*, has many DNase-encoding genes and DNase activity was found in fungal culture filtrates. The deletion of the gene for secreted DNase, *nuc1*, significantly reduced fungal infection of leaves and roots, but virulence was restored by the addition of exogenous DNase I (98). The DNase activity of recombinant Nuc1 was dependent on Mg^2+^. However, the study did not directly demonstrate that *C. heterostrophus* used extracellular DNases as a counter mechanism against extracellular DNA in ETs from plant cells.

## Parasites

### *Leishmania* spp.

The class I nuclease family has been considered an important factor for several *Leishmania* spp. to inhibit NET-mediated killing through extracellular DNA hydrolysis (99). *Leishmania* spp. have a 3’-NT/NU gene with high homology to the S1 nuclease of *Aspergillus oryzae* and P1 of *Penicillium citrinum*. The 3’-NT/NU belongs to the class I nuclease family (100), and is anchored in the parasite’s cell surface membrane (101). Although the C-terminus contains the key site for the membrane anchor domain, it was not necessary for the development of enzymatic activity. The 3’-NT/NU activity did not require N-linked glycosylation, but this modification may be important for proper folding and transport of the protein to the parasite’s surface membrane (102). The 3’-NT/NU was highly constitutively expressed and helped *Leishmania* to infect vertebrates during the life cycle (103, 104). The 3’-NT/NU had the ability to produce extracellular adenosine through 3’-AMP hydrolysis, thus contributing to the establishment of *Leishmania* infection (105, 106). Inhibition of 3’-NT/NU by 3’-AMP, 5’-GMP, or the 3’-NT/NU inhibitor, TTM, decreased the survival of promastigotes in neutrophils (47). The exonuclease activity of 3’-NT/NU was directly proportional to the number of parasites, and was more efficient at alkaline pH (48). Both low phosphate (LP) and high phosphate (HP) promastigotes induced the release of NETs from human neutrophils (47), and the NET histones killed *L. infantum.* The *L. infantum* cultured in LP medium possessed higher 3’-NT/NU nuclease activity, and LP parasites showed greater resistance to neutrophil killing. The destruction of NETs can be prevented by TTM. The hydrolysis of extracellular nucleic acids by 3’-NT/NU clearly contributed to the escape and survival of parasites exposed to NETs and promoted the establishment of infection (47, 49).

### Nippostrongylus brasiliensis

DNase II belongs to a family of highly homologous DNases that can degrade DNA to produce 3′-phosphorylated and 5′-hydroxyl products. DNase II and homologs were identified in mammals, invertebrates, and non-metazoans (107). DNase II was thought to be involved in the development and homeostasis of nematodes (108). *N. brasiliensis*, a murine hookworm, strongly stimulated neutrophils to release NETs, but the larvae secreted Nb-DNase II which degraded the nucleic acid skeleton of NETs and reduced the neutrophil killing effect. Nb-DNase II was highly expressed at the L3 stage during early infection. The extracellular DNA fibers formed *in vitro* by neutrophils induced by PMA could be hydrolyzed by the recombinant Nb-DNase II, and the degradation of NETs was neutralized by antiserum against rNb-DNase II (50). DNase II activity has also been identified in the excretory/secretory products from helminths. It is noteworthy that in comparison to other nematodes, *Trichinella spiralis* had a more extensive expansion of the DNase II protein family with an estimated 125 genes, and almost half of those were classified as encoding secretory proteins (109). Twenty-seven developmental stage-specific genes putatively encoding DNase II homologs were isolated from a *T. spiralis* cDNA library by immunological screening. In our previous work, we speculated that they may play a role in digestion of host DNA to facilitate tissue migration and escape from host immune responses (110).

### *Plasmodium* spp.

The TatD DNase was identified as one of the multifunctional factors in the development and transmission of malaria parasites. Chang et al. reported that a TatD-like DNase was present in every *Plasmodium* spp. and was highly expressed in the most virulent strains (51). The *PbTatD* deletion mutant of *P. berghei* induced J774A.1 macrophages and mouse neutrophils to generate METs and NETs *in vitro*, respectively; but, less ET network formation was observed in the presence of WT parasites. A recombinant PbTatD was able to restore hydrolysis of the DNA component of METs by a *PbTatD* deletion mutant. Interestingly, the TatD DNase activity of *P. falciparum* was inhibited by Mg^2+^, but strongly enhanced in *P. knowlesi* (52).

### *Trypanosoma* spp.

Two TatD-like DNases, TryTatD05 and TryTatD15, were identified in the supernatants of cultures of Africana trypanosome parasites, *T. evansi* and *T. brucei*, but were not released from dead parasites. *T. evansi* and T*. brucei* have been shown to induce NETs in murine neutrophils and to also be captured by NETs. However, NETs could be degraded by recombinant TryTatD05 and TryTatD15, or parasite culture supernatants (53). The degradation of NETs was significantly prevented by treatment with the DNase inhibitor, aurintricarboxylic acid. The 3’-NT/NU is a bifunctional trypanosomal enzyme, which acts as a phosphodiesterase cleaving the bond between the 3’-hydroxy and 5’-phosphoryl groups of adjacent nucleotides, or as a phosphomonoesterase to remove the 3’-terminal phosphate group of 3’-monophosphorylated nucleotides (111, 112).

## *Mycoplasma* spp.

*Mycoplasma* spp. nucleases have the ability of degradation of nucleotide substrates from host or microbial nucleic acids released in a variety of cellular processes, and several nucleases are regarded as a source of nucleotides for biosynthetic and survival purposes important and a cytotoxic factors contributing to pathogenicity (113). Intracellular, extracellularly secreted, and membrane-associated nuclease activities have been detected in most *Mycoplasma* spp. studied so far, and Ca^2+^/Mg^2+^ -dependent endonuclease activity has also been found in a comparatively large number of *Mycoplasma* spp (114). Nucleases include MGA_0676 of *M. gallisepticum*, MHO_0730 of *M. hominis*, Mhp379 and Mhp597 of *M. hyopneumoniae*, *Mbov_rs02825 and* MnuA of *M. bovis*, Mpn133 and Mpn491 of *M. pneumoniae*.

### Mycoplasma hominis

MHO_0730 is a cell-surface lipoprotein localized on the membrane of *M. hominis.* MHO_0730 shares significant homology with the Snase and acts as a Ca^2+^-dependent, sugar-nonspecific nuclease. A recombinant MHO_0730 expressed in *E. coli* was able to digest different nucleic acid substrates. PMA-stimulated NETs from human neutrophils were disassembled by *M. hominis* (54). MHO_0730 was also a potent inducer of NET formation and release through the action of the *M. hominis* liposoluble fraction and MHO_0730-based synthetic lipopeptides (55).

### Mycoplasma hyopneumoniae

Mhp597 is a secreted nuclease of *M. hyopneumoniae*. The recombinant Mhp597 (rMhp597) expressed in *E. coli* exhibited a heat stable Ca^2+^- or Mg^2+^-dependent nuclease activity. The recombinant rMhp597 induced apoptosis and caused cytotoxicity in PK15 cells. NETs induced by PMA or *M. hyopneumoniae* were completely destroyed by rMhp597, as well as culture supernatants of *M. hyopneumoniae* (56).

### Mycoplasma bovis

MbovNase is localized in the cell membrane and a recombinant MbovNase (rMbovNase) exhibited nuclease activity at 22-65°C in the presence of Ca^2+^ (115). The rMbovNase Δ181-342 without the TNASE 3 domain was deficient in all biological functions. NETs were not detected in bovine neutrophils stimulated by *M. bovis*, and NETs induced by PMA disappeared after the addition of rMbovNase. However, the degradation of NETs and *M. bovis* survival were greatly reduced by the addition of EDTA (57). MnuA was the major membrane nuclease of *M. bovis* PG45, and NET degradation was observed in WT *M. bovis*. The *MnuA* deletion mutant lacked significant nuclease activity but still stimulated bovine neutrophils to release NETs. The generation of ROS in neutrophils was not affected by the presence or absence of mycoplasma nuclease (58).

### Mycoplasma pneumoniae

Mpn491 is the main extracellular nuclease of *M. pneumoniae* and contains domains responsible for endonuclease, exonuclease, and phosphatase activities. Yamamoto et al. reported that the culture supernatants of *M. pneumoniae* had strong DNase activity and Mpn491 (approx. 55 kDa) was identified by zymographic analysis of the culture supernatant. The ability of the *Mpn491* deletion mutant to degrade NETs from PMNs was markedly abolished (59), demonstrating that Mpn491 was essential for *M. pneumoniae* to evade the NET-mediated killing *in vitro* and *in vivo*.

*Mycoplasma* spp. have certain defects in their survival process, such as a lack of ability for *de novo* synthesis of nucleotides. *Mycoplasma* spp. secrete nucleases to obtain nucleotides from host genomic DNA to compensate for this defect. MET production induced by PMA could be observed in THP-1 cells, and was degraded by the membrane nucleases from *M. hyopneumoniaea*. MET degradation was inhibited by deficiency in *M. hyopneumoniae* nucleases. The nucleotides from MET degradation can be used for DNA synthesis (116). Therefore, nucleases are widely considered to be essential proteins for *Mycoplasma*, and also have been proven to be important virulence factors during infection.

## *Leptospira* spp.

It has been shown that pathogenicity and viability of *Leptospira* spp. were relevant factors for induction of NETs, but not for motility. A pathogenic *Leptospira*, *L. interrogans* serovar Copenhageni strain Fiocruz L1-130, induced human neutrophils to release NETs, which were capable of killing *L. interrogans* (60). The pathogenic *Leptospira* showed DNA degradation activity, but not the saprophytic *Leptospira*. *Leptospira* immunoglobulin-like proteins (Lig) are surface proteins expressed in pathogenic strains of *Leptospira*, including LigA, LigB, and LigC. The Lig proteins were involved in leptospiral adhesion, complement resistance, and modulation of hemostasis and play a vital role in invasion and immune evasion (117).The recombinant variable region of LigA was able to degrade DNA with both endonuclease and exonuclease activities, and could degrade PMA-induced NETs (118).

## Conclusions and Perspectives

Since their discovery in 2004, ETs have been described from a wide variety of innate immune cells and are now widely regarded as an ancient and evolutionarily conserved host defense mechanism in the plant and animal kingdoms (119, 120). Several pathogens can produce one or more nucleases to directly act on the DNA skeleton of ETs (6). In this review, we summarized the current study results on nucleases expressed by pathogens to evade the ETs produced by the host immune system. The main concerns are 1) only certain pathogen-derived nucleases degrade ETs. Pathogens included *V. cholerae*, *Streptococcus* spp., *S. aureus*, *M. tuberculosis*, *P. aeruginosa*, *N. gonorrhoeae*, *P. intermedius*, *R. solanacearum*, *Leishmania* spp., *N. brasiliensis*, *Plasmodium* spp., *Trypanosoma* spp., *Mycoplasma* spp., and *Leptospira* spp. 2) types of nucleases. Thirty-four nucleases have been reported to be associated with ET degradation, and among them, eleven were exonuclease/endonuclease and twenty-three were categorized as DNase, sugar nonspecific nuclease, or nucleotidase. 3) with regard to the cellular localization of nucleases in pathogens. The formation of ETs occurs in the extracellular space of immune cells, and secreted extracellular nucleases from pathogens are the most direct way of degradating the extracellular networks. The nucleases may be anchored on the cell wall, the cell-wall surface, the membrane, or the cell-surface membrane; and parasitophorous vacuole membranes and surface proteins also have the ability to degrade ETs. 4) only ETs from certain species and cell types were used to investigate to the degradation of ETs by pathogen-derived nucleases. Neutrophils were initially used as the cells for producing and studying ETs, and considering the similar network structures of NETs and ETs from other immune cells, current research has mostly focused on the nucleases that hydrolyze the DNA of NETs. Therefore, for a number of other ETs, the role of nucleases still needs to be evaluated.

Future research will most likely focus on designing drugs to neutralize the nuclease activity of pathogens to preserve the integrity of host NETs and combat invading organisms. The protein synthesis inhibitor clindamycin and the immunoglobulins efficiently inhibited the nuclease activity of *S. aureus* by reducing the transcription of *nuc1*, resulting in enhanced NET-mediated extracellular clearance in human blood-derived neutrophils (121). The antimicrobial peptide (AMP), LL-37, a member of the cathelicidin family, facilitated the formation of NETs by human blood-derived neutrophils (122). Interestingly, LL-37 effectively prevented the nucleases of *S. aureus*, *S. pneumoniae* and GAS from degrading NETs (123), as well as the cationic AMPs, human β-defensin-3 and human neutrophil peptide-1.

ETs are thought to be dismantled by the secreted nucleases, DNase I and DNase1-like three protein (DNase1L3, also known as DNase γ) in host (124). The fragments or remnants are removed by macrophages and dendritic cells, and the process was dependent on the cytosolic exonuclease TREX1 (also known as DNaseIII) and extracellular DNase1L3, respectively (125). It is worth noting that uncontrolled NET formation or insufficient NET removal can cause highly detrimental effects to host cells *in vivo* such as cell damage, delay in tissue repair, inflammation, vaso-occlusion, autoantibody production, tumor capture, tumor growth, cytokine, and chemokine degradation (126). The crucial role of NETs in the pathogenesis of metabolic diseases, sepsis, thrombosis, autoimmune and autoinflammatory diseases, cancer, and other human diseases has been extensively studied and reviewed (127). The administration of DNase to dismantle ET structures can ameliorate disease progression in mouse models of breast cancer, lung injury, and systemic lupus erythematosus (128–130). Dornase alfa (Pulmozyme^®^, recombinant human DNase) is currently used in the clinic to treat pulmonary disease in CF (131). Dornase alfa treatment reduced the amount of NETs in the respiratory tract, leading to less airway obstruction in severe bovine RSV infection (132). Therefore, pathogen-derived nucleases should be considered as the treatment of choice for ET-mediated diseases in the future.

On the one hand, the massive amounts of ETs degraded by nucleases *in vivo* are helpful for preventing the occurrence of cardiovascular, immunological, and metabolic diseases, and cancer (133). On the other hand, some pathogens such as *V. cholerae*, *S. aureus*, *P. intermedia*,and *Actinobacillus pleuropneumoniae* (*A. pp*) may benefit from circulating cell-free DNA, adenosine, and NAD from degraded NETs as a source of nutrients. For example, *A. pp* did not produce extracellular DNases, whereas porcine NETs were efficiently degraded by nucleases from *S. suis* and the products could be used as an external NAD source for *A. pp* growth when co-infecting *S. suis* (134). Therefore, more studies are needed in the future to unambiguously determine the relationship between ET formation and degradation by pathogen nucleases or host DNases (135, 136).

## Author Contributions

CL conceived the idea and guided the whole work, and wrote the draft. FM and MQ searched the literature and wrote the draft. MQ and XW revised the manuscript. All authors read and approved the final manuscript.

## Funding

The review was supported by the National Natural Science Foundation of China (32102705 and 31802159) and the youth backbone teachers training program of Henan University of Science and Technology (13450009).

## Conflict of Interest

The authors declare that the research was conducted in the absence of any commercial or financial relationships that could be construed as a potential conflict of interest.

## Publisher’s Note

All claims expressed in this article are solely those of the authors and do not necessarily represent those of their affiliated organizations, or those of the publisher, the editors and the reviewers. Any product that may be evaluated in this article, or claim that may be made by its manufacturer, is not guaranteed or endorsed by the publisher.
